# Development of Preliminary Precision Forging Technology and Concept for Tools Used to Reforge 60E1A6 Profile Needle Rails with the Use of Numerical and Physical Modeling

**DOI:** 10.3390/ma16052103

**Published:** 2023-03-05

**Authors:** Marek Hawryluk, Piotr Cygan, Jakub Krawczyk, Artur Barełkowski, Jacek Ziemba, Filip Lewandowski, Igor Wieczorek

**Affiliations:** 1 Department of Metal Forming, Welding and Metrology, Wroclaw University of Science and Technology, Lukasiewicza Street 5, 50-370 Wroclaw, Poland; 2 Kuźnia Łabędy S.A., Mechaników 9, 44-109 Gliwice, Poland

**Keywords:** forging technology, rail ends, closed die, numerical and physical modeling, die cavity

## Abstract

This study examines the possibilities of applying numerical and physical modeling to the elaboration of technology and design of tools used in the hot forging of needle rails for railroad turnouts. First, a numerical model of a three-stage process for forging a needle from lead was built in order to develop a proper geometry of the tools’ working impressions for physical modeling. Based on preliminary results of the force parameters, a decision was made to verify the numerical modeling at 1:4 scale due to forging force values as well as agreement of the numerical and physical modeling results, which was confirmed by the similar courses of forging forces and a comparison of the 3D scan image of the forged lead rail with the CAD model obtained from FEM. The final stage of our research was modeling an industrial forging process in order to determine the preliminary assumptions of this newly developed method of precision forging using a hydraulic press as well as preparing tools to reforge a needle rail from the target material, i.e., 350HT steel with a 60E1A6 profile to the 60E1 profile used in railroad turnouts.

## 1. Introduction

**Figure 1:**
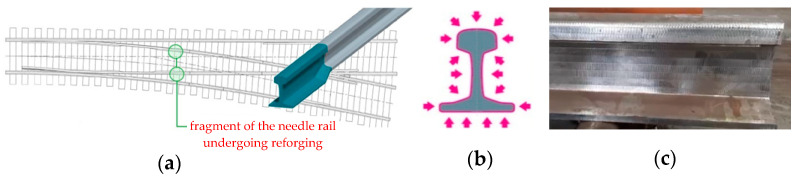
View of (**a**) a fragment of a railroad turnout with the marked needle rails subjected to forging, (**b**) a CAD model with the marked surfaces of the cross section necessary for the mechanical treatment, and (**c**) an image of a mechanically treated reforged needle rail as a result of the previous underdeveloped die forging technologies.

Needle rails are railway infrastructure elements that are fixed in pairs in turnouts so that the train’s track or travel direction can be changed (Figure 1). On one side, they have a so-called facing point, which is made from a needle rail through mechanical processing, whereas on their other end, as a result of plastic treatment, the needle rail is reforged into a typical rail with a regular profile, which is next connected to the remaining part of the turnout in a thermite welding process [1].

Currently, the processes of needle rail reforging are realized mainly through semi-closed die forging or die forging, as a result of which the obtained reforged parts of the needle rail are characterized by improper geometry, causing the necessity of large-scale mechanical treatment, as well as insufficient hardness and low surface quality. For this reason, the technologies realized so far require improvement and optimization, as they necessitate the production of forgings with big allowances for mechanical treatment on all surfaces of the rail (Figure 1c). This mechanical treatment is not only time-consuming and expensive [2], but also significantly lowers the strength properties, especially fatigue strength at lower temperatures, which is very important for train rails, which undergo cyclic loads under extreme conditions, especially during the passage of large heavy freight train sets [3,4]. This causes the production of needle rails for railroad turnouts to be very costly and time-consuming. However, continuous attempts have been made to optimize the current technologies, yet the technology of precision forging of needle rails is still not very well known and developed [5]. The elaboration of a new precision forging technology that would significantly eliminate mechanical treatment is a chance to solve many problems and thus strengthen the production and improve the quality and interoperability of railroad infrastructure [6]. It should be mentioned that, apart from developing new precision forging technologies, an important aspect is also the wear of the rails in turnouts, especially during high-speed train passages, as this can seriously affect the safety of train travel as well as the durability of the wheels and the turnouts. For this reason, at present, we can observe a new direction of development in this area, i.e., the application of bainitic steels, which ensure long operation and are dedicated for high-speed trains [7,8], which have separate performance issues.

The correctness of all die forging technology is dependent on many, often opposing, factors as well as their interaction, which depend mainly on the technology itself and the operation conditions, the type of the forging and its material, and also, often, on the human factor [9,10]. All this makes die forging processes difficult to analyze and optimize, while at the same time constituting one of the most difficult industrial production processes. For this reason, in order to design, analyze and improve technologies based on plastic forming of materials, a whole spectrum of CAD/CAM/CAE/FEM methods is applied [10,11,12,13] as well as other IT tools (information technology tools) [14,15,16] and physical simulations, often combined with numerical modeling [17,18]. This is because developing a proper industrial process of plastic treatment requires many experiments and tests on the real material, which is connected with huge costs and large time input, and still, the most important stage of design and optimization is the final verification of the elaborated process under industrial conditions [19,20]. Therefore, there is a constant search for new scientific methods that not only facilitate the fast and precise design and analysis of plastic forming processes, but also eliminate the need for experiments on real material or trials under industrial conditions as the final verification. For this reason, currently, studies are conducted in two main directions. The first is based on new calculation techniques applying numerical modeling and IT tools [21], which make it possible to develop mathematical models of different plastic forming processes and phenomena occurring in the deformed material [22,23,24,25] or provide new methods and tools enabling a partial replacement of time-consuming and expensive experiments by so-called virtual experiments. The other main direction is experimental methods based on simulations and physical modeling, often with the use of model materials [26,27]. Despite the unquestionable justification of the implementation of numerical modeling and tools based on artificial intelligence in the area of plastic forming processes, we should also take into consideration certain limitations of these methods [28]. The main limitation of the use of techniques based on a mathematical apparatus (FEM, IT) in the designing process is the correctness of the obtained results. Uncertainty can be caused by wrong assumptions, an improper model or calculation errors, which make the obtained results possibly burdened with error of up to 10%. Although numerical modeling and IT tools significantly change the role and scope of experiments into the virtual dimension, the real experiment remains the most expensive and time-consuming stage of design and optimization [29,30]. For this reason, an alternative for numerical modeling and IT tools can be physical modeling methods with the use of soft model materials (mainly based on plasticines) as well as the commonly applied metallic model material lead (Pb), owing to which it is possible to reduce the costs of the real experiment. These methods, as ones that are much cheaper, faster and easier, can constitute another direction in the development of methods aiding the analysis and design of plastic forming processes as well as play a verification role for first directions or industrial processes themselves. Physical modeling methods can also be an independent tool in the design and analysis of plastic forming processes with the consideration of both the shape and the properties of the ready product, or they can cooperate with numerical modeling, thus providing a lot of necessary information relating to the behavior of the deformed material or the initial boundary conditions.

The process of forging turnout needle rails consists in preliminary heating of a fragment (about 1 m) of the rail to over 1200 °C, and next reforging the end of the needle rail, on a slightly shorter distance than the heated one, into a track rail. At present, the realized forging processes are conducted usually in a few semi-closed die forging impressions, and sometimes, the last operation is one of die forging [31,32]. In most cases, additional inter-operation heating is applied, which can cause a rapid increase in decarburization degree on the surface of the produced needle rail elements, as well as significantly prolong production time and increase unit production costs. Additional inter-operation heating usually results from insufficient accuracy of the geometry of the forging tools’ working impressions, as well as insufficient pressures in the forging aggregates, precision of transport and manipulation of the rails [33]. In the technologies realized so far, other important limitations include obtaining a homogeneous microstructure and mechanical properties in the whole needle rail and reforged area, especially in the so-called soft spot present in the contact area between the part heated for forging and the non-formed part, as well as difficulty in the thermal treatment of new pearlite and bainitic rail steels, which were introduced into the European railway market relatively recently [34,35,36,37]. All this means that, during times of high energy prices and the broadly understood need to protect the natural environment, these technologies require further development in the technical-technological aspect [38,39]. Elaboration of a precision forging technology involves solving a series of problems and issues in terms of plastic treatment, materials engineering and automatization, and control of the production processes [40]. We should, however, clearly point out that the available literature and other publications provide hardly any information on the technology of needle rail reforging, not to mention precision forging.For this reason, in the analysis of the currently realized processes of needle rail forging, while simultaneously considering the development of needle rail precision forging technology, the most important technological challenge is undoubtedly the proper design of tool (die) impressions in order to obtain such a reforging shape that will minimize the needed mechanical treatment to as close to zero as possible. Additionally, all the above should happen with the consideration of the available forging aggregate and no need for inter-operational heating. For this reason, the best solution that will make it possible to achieve such a goal is a simultaneous application of numerical modeling and physical modeling as mutually supplementary methods replacing the real experiment—the industrial process of production.

## 2. Test Subject and Methodology

The aim of this study is the conceptual development of needle rail reforging tools that will make it possible to realize precision forging technology under industrial conditions with the use of mainly physical and numerical modeling as well as other scientific and measurement methods. In order to reach the assumed objective, the following research studies were conducted, including 3 main stages:

- Numerical modeling with the use of the Forge 3.0 Nxt calculation package for the process of reforging a rail section for the model material (lead), not for the target material, i.e., steel R350HT. The simulations were performed for such a material because the following step of research was verification of the obtained results based on physical modeling.

The calculations were made at 1:4 scale as the preliminary FEM simulation results for Pb indicated that, in the case of the real size (dimensions) of the tools, the forces would exceed the maximal pressure of a semi-industrial hydraulic press, 200 t;

- Physical modeling of the process of forging a needle rail from lead in order to verify the virtual forging process (numerical modeling). A justification of performing physical modeling at a reduced scale is the facility and relatively low costs of such an experiment, as well as the fact that the large mass of forging (for the real dimensions) would cause problems with transport and the manipulation and removal of tools;
- Analysis of the obtained results, numerical modeling of the industrial forging process and elaboration of the target tools (with the consideration of the real dimensions of forging) used to forge needle rails from R350 HT pearlitic steel.

## 3. Tests and Discussion of Results

### 3.1. Numerical Modeling

**Figure 2:**
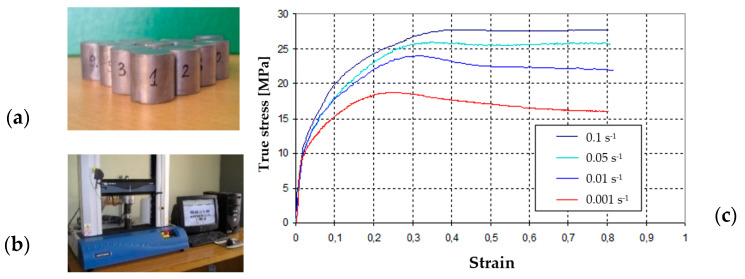
View of: (**a**) a set of samples for compression tests, (**b**) an image of the test station, (**c**) the flow curves for Pb.

In the first place, in order to design tools for physical modeling at scale, numerical modeling for the process of reforging a rail section from Pb. The scale of 1:4 was selected, as preliminary simulation results indicated that for 1:2 scale, the forces exceeded the maximal press pressure. The choice of lead as the model material was dictated by the fact that it is a commonly used metal that very well simulates the behavior of hot deformed steel [26,41], and additionally, it is relatively cheap and easy to prepare (through casting into a die and possibly re-forming). Based on the performed multi-variant simulations considering different numbers of forging operations as well as different shapes of the particular impressions, it was decided that a process of 3 operations would be modeled. Such a tool geometry variant was selected that would ensure the lowest force values and proper filling of the impressions as well as lack of defects in the forms of laps or underfills. The following boundary conditions were assumed in the simulation: initial temperature of the reforged Pb rail section—22 °C, tool temperature—22 °C, ambient temperature—25 °C, press speed—22 mm/s, friction coefficient—0.2, thermal conductivity at contact—25 kW/m^2^ K, thermal conductivity with the environment—15 W/m^2^ K. In addition, with the known parameters of the target forging aggregate, a hydraulic press with a pressure of 3000 t and a maximal movement speed of 30 mm/min, as well as the preliminary assumptions of the elaborated technology, for the target process, it was assumed that the mean deformation rate would be at the level of 1 s^−1^. In the modeling process, the assumed press speed was at the level of 22 mm/s. The flow curves for lead determined in a trial of upsetting a cylinder (d = 20 mm, h = 30 mm) on an Instron testing machine were introduced into the calculation program in a tabular form (Figure 2).

**Figure 3:**
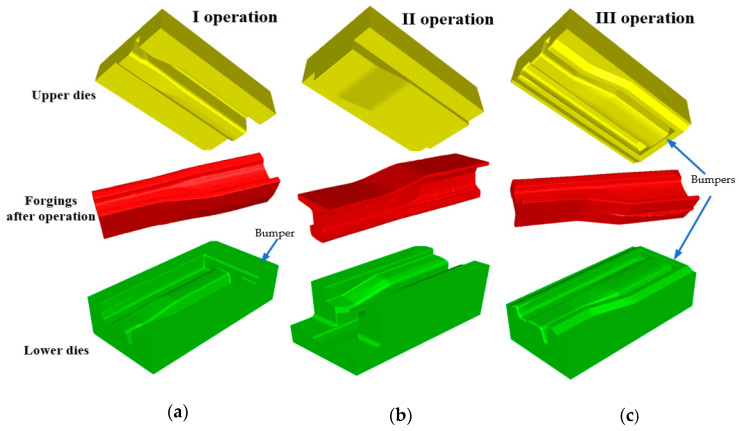
Final versions of CAD models of tools used to reforge needle rails from Pb at 1:4 scale: (**a**) for operation I, (**b**) for operation II, (**c**) for operation III.

Figure 3 shows the elaborated final versions of CAD models of tools for reforging needle rails from Pb at 1:4 scale that were implemented into the Forge 3.0 NxT program. The designed tools consist of: operation I—top and bottom, operation II—left, right and top, operation III—top and bottom. Additionally, the conducted simulations also considered additional forming elements (stops) in operations I and II, which aimed at ensuring filling of the impressions as well as the lowest force values.

**Figure 4:**
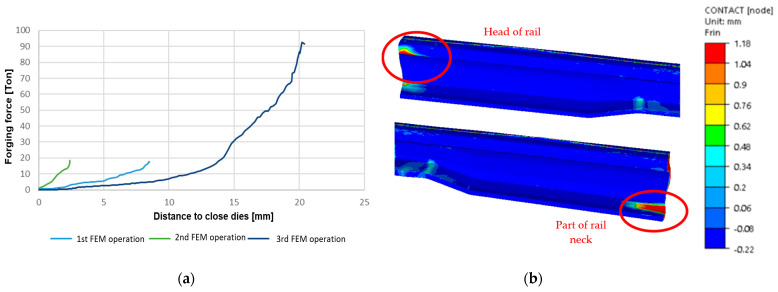
View of: (**a**) the forging force courses for the particular operations, (**b**) the forging defects in the form of underfills on the foot and the head of the reforged rail.

Figure 4 presents the course of forging forces for the particular forming operations of a 60E1A6 needle rail at ¼ scale from lead, as well as the occurring problems with filling the impression in operation III in the head and the inside part of the foot (Figure 4b).

**Figure 5:**
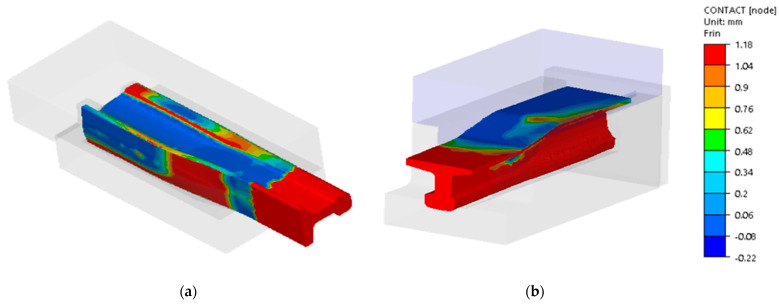
Results of contact for forging: (**a**) after operation I, (**b**) after operation II, blue colour means full contact between forming material and tool.

As we can notice, the highest value of forging force (Figure 4a) occurred in operation III and equaled about 91 t. Due to this fact, there was no risk of exceeding the acceptable pressure of the semi-industrial press 200 t. After applying the mentioned stops and slightly smaller allowances, it was possible to obtain proper filling of impressions in the following forging operations (Figure 5a,b). In turn, in the analysis of the results of impression filling in operations I and II, proper filling was obtained. However, in operation II, in the area of the neck and the head (Figure 5b), we can notice a small distance between the stop and the forging with a value below 1 mm. What is more, a certain clearance should be maintained in this area so that the reforged element can be removed and transported to operation III.

**Figure 6:**
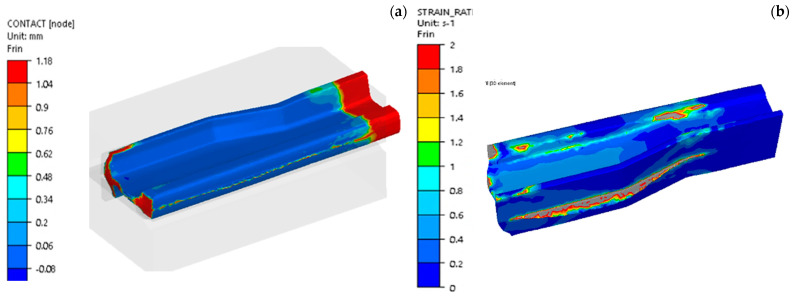
Results of the FEM simulation: (**a**) contact for forging after III, the final forging operation, (**b**) the mean deformation rate.

In operation III, filling is the key factor, as the developed technology should ensure that, after the forging process, only mechanical treatment of the feet and the head of the rail need to be performed, whereas the neck should be ready-made. For this reason, a detailed analysis of the filling was performed for operation III (Figure 6), and additionally, the mean deformation rate in this operation was determined in order to select the parameters of the model material, Pb.

**Figure 7:**
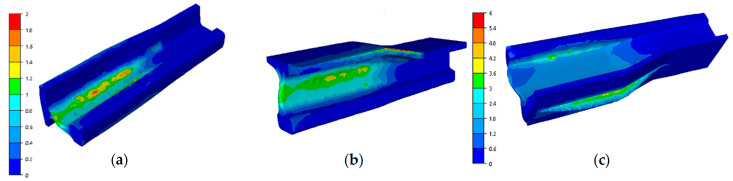
Simulation results—plastic deformation distributions: (**a**) after forging operation I, (**b**) after forging operation II, (**c**) after forging operation III.

The results presented in Figure 6 of the filling in the last step of the final III operation, that is, reforging a re-scaled needle rail from lead 60E1A6 to 60E1 with reforging length of 75 mm show a proper way for material to flow and fill. We can notice local underfills, which, unfortunately, cannot be avoided, as they are a result of the introduced limitations in terms of the dimensions of the rail and the tools participating in the tests. Longer dies would limit the rail material flow along the length, which would improve the filling, but they would also increase the deformation force. In an industrial process, such an underfilled end is cut off. In the case of the results of the deformation rate distributions in operation III, we can assume that they were at the level of 1 s^−1^, which also corresponds with the assumed deformation rate for R350HT steel in the target industrial press. For a more thorough analysis, the plastic deformation distributions were determined for the operations, which are presented in Figure 7.

As we can notice, the plastic deformations in the first and second operation are concentrated mostly in the neck and partially in the foot and equal about 2. In turn, in the third operation, the highest deformations occur on the head and in the flash on the foot of the reforged rail. Such a distribution of deformations is in agreement with the developed technology, in which the tools were designed in such a way so that, in operation I, the rail neck is formed, in operation II, the neck undergoes elongation and reforming and shaping of the foot begins, and in operation III, shape calibration of the whole rail takes place.

### 3.2. Physical Modeling from Pb

**Figure 8:**
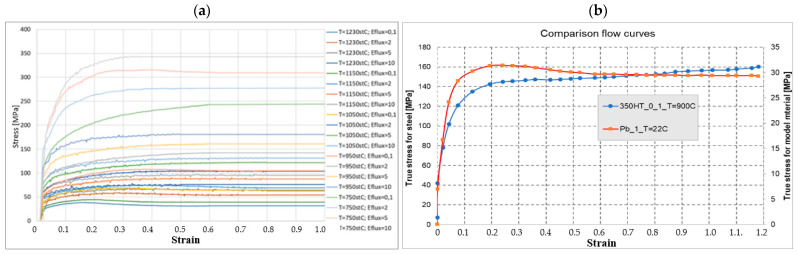
Selection of the model material Pb for R350HT steel: (**a**) the flow curves for R350HT steel, (**b**) a comparison of the flow curves for selected dominating deformation conditions.

In order to confirm the selection of the model material, Pb, for steel R350HT, the description of material similarity in the plastic scope proposed in [42] was applied. It assumed a quantitative assessment of the matching degree of the shape of the yield stress–deformation curves for the model material and the actual material. Two parameters were determined: the scale factor **C** and the similarity coefficient **t**. The coefficient *t* is a non-dimensional quantity that enables a simple and easy determination of the matching degree of the reinforcement curve of the model material to the real material. In the case of a theoretically ideal matching of both curves, this coefficient should equal zero. A detailed description of the condition and its verification can be found in a few studies [42]. The use of lead as the model material was made mostly for the determination of the flow manner and the filling of working impressions as well as owing to the facility of performing a physical modeling experiment. The reinforcement curves of R350HT steel were determined experimentally in a universal simulator of metallurgical processes, the Gleeble 3800 system, for 4 deformation rates (0,1; 2; 5; 10 1/s), at 5 temperatures (750; 950; 1050; 1150; 1230 °C), in a deformation scope from 0 to 1 (the value of deformation was limited by the sample and the upsetting test) (Figure 8a). The performed preliminary numerical simulations of the elaborated forging technology for an industrial process on simplified tools indicated that, for most of the process, the temperature of forging was within the scope of 850–950 C, and the deformation rates were at the level of 0.5–2 s^−1^. For this reason, the similarity condition in the plastic scope was used to determine the scale factor and the similarity coefficient for the predetermined deformation rate for 350HT steel; it was decided that the most important aspect for the course of the process was the material flow curve for the temperature of 900 °C and the deformation rate of 1 s^−1^ [4]. Therefore, on this basis, the selected flow curve was for the ambient temperature for Pb and the same deformation rate, equaling 1 s^−1^. The obtained results are presented in Figure 8.

As we can see, the shapes of the flow curves for R350HT steel and lead for the deformation rate of 1 s^−1^ are similar. With use of the equations from the plastic similarity conditions, the obtained value of factor C was at the level of 6 and the similarity coefficient equaled 0.05. The presented results according to the plastic similarity conditions are highly satisfactory, as they demonstrate, with a relatively small error, that lead quite well simulates the behavior of real steel material, especially in the aspects of flow and the proper filling of impressions.

**Figure 9:**
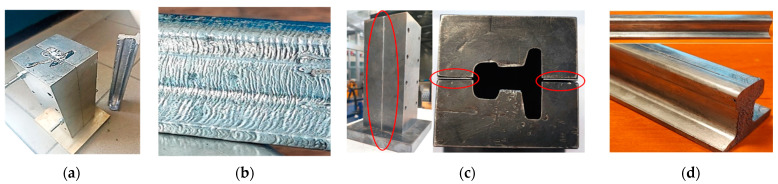
Images of: (**a**) the mold for casting a 60E1A6 needle rail from lead at ¼ scale, (**b**) a poor-quality needle rail surface after casting, (**c**) the mold with fixed spacers increasing the forging bay’s volume, (**d**) the rail after preliminary forming.

The subsequent stage of research was designing and building of the mold (re-scaled 1:4) to cast a 60E1A6 type needle rail (Figure 9a).

The first trials of casting Pb into the mold did not give the desired results (Figure 9b), as the surface of the rail was very corrugated and rough, which was a result of too-rapid cooling of the lead in the mold. For this reason, in order for the rail surface to be smooth, spacers aiming at increasing the mold’s bay were used (Figure 9c), and next, the spacers were removed and the mold with the lead was compressed inside on a press with the force of 100 t, thus obtaining a high-quality surface (Figure 9d).

**Figure 10:**
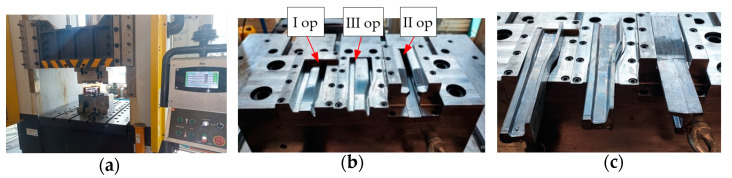
Images of: (**a**) the physical modeling station, (**b**) the lower tools for three operations of reforging a lead rail, (**c**) the formed rails placed in the lower dies.

Figure 10 shows the work station and the prepared tools for physical modeling. From the left, we can see the lower forging tools in the order I, III (last forging operation) and II (forming operation) of a re-scaled lead rail. The prepared needle rail preforms were reforged (reformed) on a section of 75 mm into shape 60E1 (physical modeling at 1:4 scale). A total of 6 reforging tests were performed, from which very similar results were obtained.

**Figure 11:**
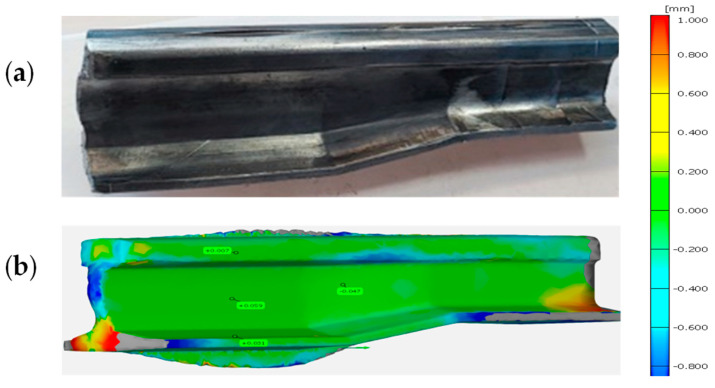
Results: (**a**) a photograph of the lead rail after the last forming operation, (**b**) a comparison of the shape and dimensions from FEM with the experiment, with a map of deviations (green color is best fit).

To verify the physical modeling, we compared a 3D scan of the reforged needle rail made of Pb to the nominal CAD model, which is shown in Figure 11.

As we can observe based on the 3D scanning results, the obtained geometry from the laboratorial process is in good agreement with the numerical modeling, and errors of shape, even in extreme areas, do not exceed 1 mm.

**Figure 12:**
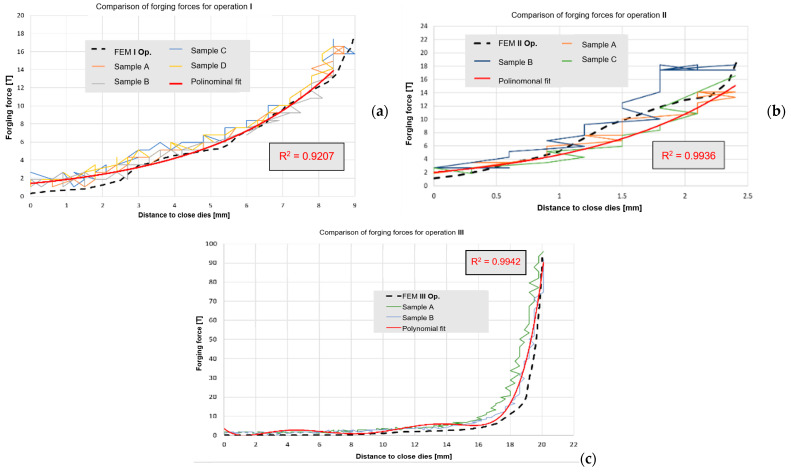
Comparison of the forging forces for each of the 3 operations of physical modeling with the FEM results: (**a**) operation I, (**b**) operation II, (**c**) operation III.

The following stage of verification of the correctness of the FEM calculations was a comparison of the forging forces determined from the numerical modeling with the forces measured during each of the 3 operations of forming a 60E1A6 rail from lead (Figure 12a–c).

We can notice that the results calculated numerically are similar to the real values resulting from the measurements (the force results from the press could only be read out at certain time distances, and so they fluctuate). Additionally, for a selected sample, for each of the 3 operations from physical modeling, an equation was formulated and the error with respect to the forces obtained from FEM was determined. As we can see, for each case, the difference is not more than 8%. This allows us to state that the elaborated numerical model for forging a lead rail is correct. To verify, let us assume yield stresses for Pb at the levels of 28 MPa and 158 MPa for R350HT steel formed in the last operation with a forging temperature of about 900 °C. For them we obtain scale factor C at the level of 158:28 = 5.6. Next, if we assume that the maximal force in the process occurs in the last operation, III, then for the forming of Pb, according to the diagram, it is at the level of 108 t. Therefore, by multiplying 108 × 5.8 and considering the scale factor of ¼, we obtain 108 × 5.6 × 4 = 2419 t, that is, theoretically, such a value of force should be expected in the last operation of needle rail forging in the industrial process for R350HT steel. Therefore, this confirms the maximal load of the selected hydraulic press with a pressure of 3000 t, and the obtained results can be used to develop a proper model for the target forging process for 350HT steel. The correctness of physical modeling using the geometric scale of enlarging/reducing the physical model to the real one was demonstrated in [43]. However, we should note that, the matching of the model material Pb to 350HT steel equaled 0.054, which, according to the proposed material’s plastic similarity in the plastic scope, shows that such a material well simulates the manner of flow of the real material. In turn, the force parameters can be burdened with error. What is more, we should be aware of the certain simplifications applied in physical modeling, as the mean deformation rate and temperature that occurred in the numerical modeling of the target process were assumed mostly on the basis of the results for operation III, in which the highest values of forming forces were expected. Nonetheless, such an approach is justified for the purpose of minimizing the costs of the industrial experiment and undertaking an attempt to elaborate the target technology under industrial conditions with the greatest probability of success.

### 3.3. Numerical Modeling of an Industrial Forging Process Based on the Results of Numerical Modeling for Lead at 1:4 Scale Verified by Physical Modeling

**Figure 13:**
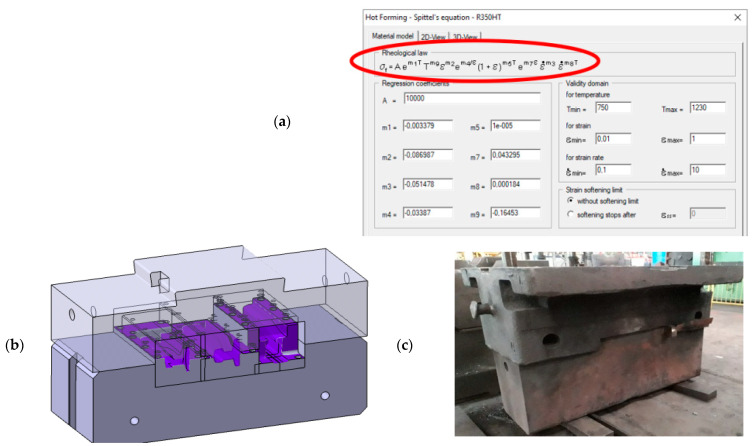
View of: (**a**) a screen from the program with the Spittel equation and the determined coefficients, (**b**) the CAD model of the particular tools fixed in the anvils, (**c**) a photo of the lower holder for reforging needle rails before modernization.

The obtained physical modeling results confirm the correctness of the assumptions made in numerical modeling. Therefore, on this basis, a decision was made to re-scale the geometry of the elaborated tools (together with the working impressions) for the process of physical modeling from Pb into the real tool dimensions, which were imported into the FEM program. Next, a numerical model was built with the following assumed initial-boundary conditions: initial temperature of the reforged rail section—1280 °C, tool temperature—280 °C, ambient temperature—30 °C, press speed—22 mm/s, friction coefficient according to the Coulomb model—0.15, thermal conductivity in contact—10 kW/m^2^ K, thermal conductivity with the environment—10 W/m^2^ K. The strength properties of the forging material—the flow curves of R350HT steel (Figure 7a)—were introduced into the program by way of a non-linear estimation of the coefficients in the Spittel equation. This equation was introduced into the Forge program as the material data describing the mechanical properties of R350HT under hot forging conditions (Figure 13a).

The rail reforging simulations were performed for a process that was ultimately planned to be realized under industrial conditions on a hydraulic press with maximal pressure force of 3000 t. The assumed rail reforging length was at the level of 240 mm, with a 60 mm allowance to be cut off; the total reforging length was 300 mm. It was also assumed that the anvils supporting the die inserts would be 600 mm long (Figure 13b), and so, it would also be necessary to reconstruct and modernize the currently applied holders fixed on the press (Figure 13c). Additionally, it was assumed that transporting the rail between operations would last up to 30 s (such boundary conditions were applied in the numerical calculations). The presented changes of the forging conditions made it necessary to conduct a numerical simulation of this process. In the calculations, the tools for all the forming operations were modified and the times of transporting the forging onto the dies for the consecutive forming operations were prolonged, which was connected with the planned manual manipulation of the rail during the process.

**Figure 14:**
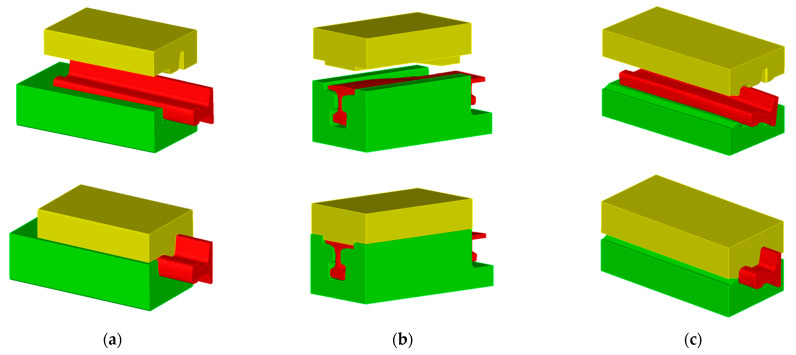
General view of the numerical model before and after: (**a**) operation I of reforging a 60E1A6 rail, (**b**) operation II, (**c**) forging operation III.

The constructed numerical model before the forming of the rail and in the last stage of operation I is presented in Figure 14a. In this operation, a flash is not formed. The lower die was closed on one side, which showed how deep the rail should be placed (to create a so-called stop) and how to inhibit the material flow in this direction. In turn, Figure 14b shows a general view of the numerical model before the rail forging in operation II and in its last step.

**Figure 15:**
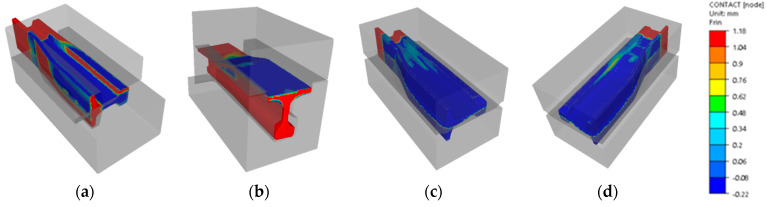
Contact distribution after: (**a**) operation I, (**b**) operation II, (**c**) operation III (top view), (**d**) operation III (bottom view); the contact area is marked in dark blue.

In turn, Figure 15 shows the results of numerical calculations for a 60E1A6 needle rail in the form of contact of the forging with the dies’ working surfaces.

**Figure 16:**
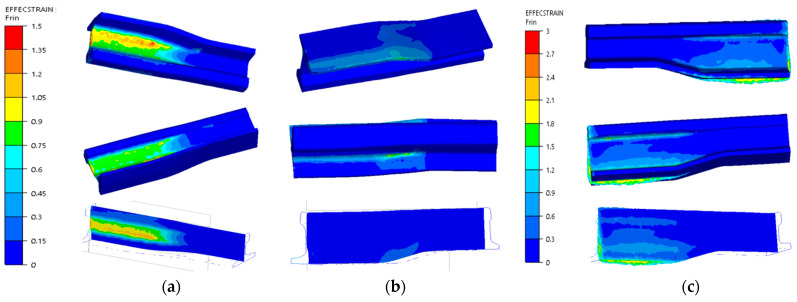
Deformation intensity distribution after the consecutive forging operations: (**a**) after operation I, (**b**) after operation II, (**c**) after operation III.

For a more thorough analysis, Figure 16 presents the plastic deformation distributions after the consecutive operations. The highest deformation values in the first operation (Figure 16a) occurred in the middle part (between the head and the foot) in the area of the rail’s contact with the upper tool and locally equaled about 1.5. In the remaining areas, the deformation intensity was much lower. On a half-section, the deformations equaled about 1–1.2. In turn, Figure 16b shows plastic deformations for operation II, which do not take into account the deformations in forging operation I, as dynamic recrystallization took place between these operations. The plastic deformations assume the highest values in the area of the flash (in the allowance area on the foot) as well as in the area of the rail’s radius of transition into the central part, as in the case of forming a rail with the whole reforging length. In this forming operation, the rail was almost not deformed inside on the cross section. In turn, in Figure 16, we can see the plastic deformation distribution after 3 forming operations. Between stage 2 and 3 of the forming process, the rail material also underwent dynamic recrystallization, and so, these were the only deformations brought about during operation III. We can notice that the highest deformations were in the vicinity of the flash—their values reached about 3.

**Figure 17:**
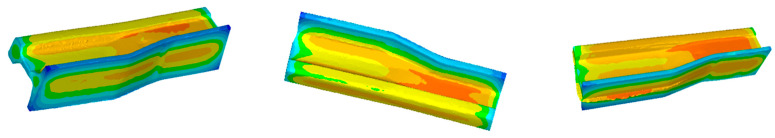

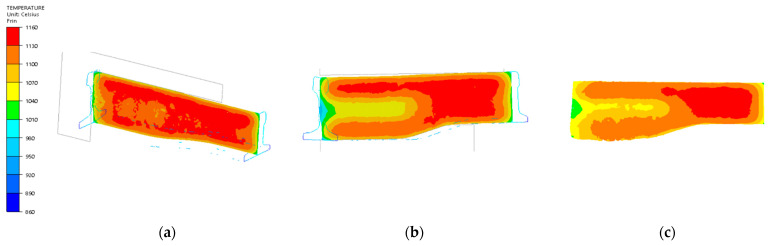
The temperature distribution after the consecutive forging operations: (**a**) after operation I, (**b**) after operation II, (**c**) after operation III; the lower results are for the cross sections of the reforged rails.

An analysis was also performed of the temperature field distributions in the forging after the consecutive operations (Figure 17). Figure 17a shows the temperature distributions on the surface and inside the forging in the last simulation step of forging operation I. The temperature dropped most on the corners of the forging—to about 930 °C. Inside the forging, the temperature also decreased, yet only to about 1210 °C. We can notice that the prolonged time of transporting the forging from the furnace to operation I caused a decrease in the rail’s temperature to about 70–250 °C, which had an effect on the forming force.

**Figure 18:**
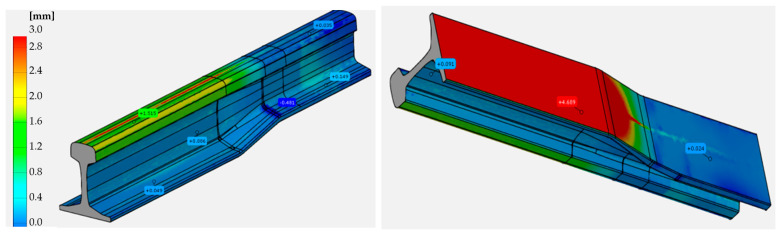
Distribution of deviations of the shape calculated from FEM from the nominal shape and dimensions from the CAD model.

In the case of operation II (Figure 17b), the temperature in the reforged rail dropped on the corners to about 860 °C, and on the cross section, inside the rail, to about 1150 °C. In turn, in operation III (Figure 17c), the temperature on the corners decreased to about 730 °C, and on the cross section, inside the rail, to about 1100 °C. The big temperature drop is connected with the increased time of the rail’s transport between the operations. Figure 18 shows a comparison of the shape and dimensions of a 60E1A6 rail calculated numerically against a CAD model.

We can notice that the allowances for the trimming treatment are in the correct areas, the shape and dimensions of the rails correlate quite well, and the determined deviations are at the level of 0–1.5 mm, that is, they are within the assumed tolerance field.

**Figure 19:**
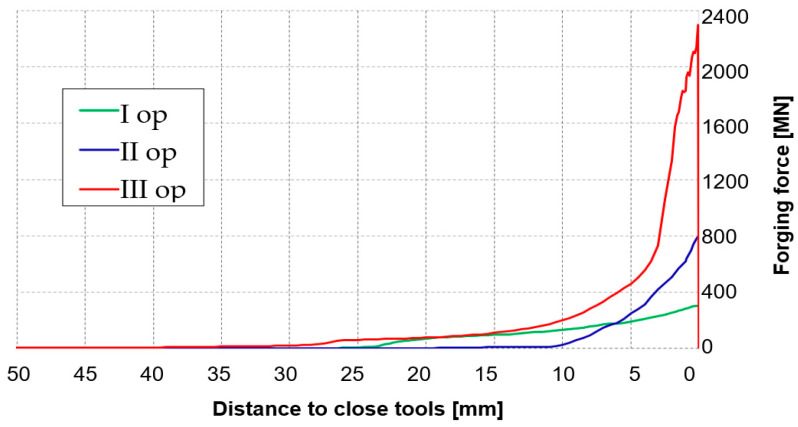
Courses of forging forces in operations I, II and II of forming a 60E1A6 rail.

Figure 19 shows a diagram of the forging forces in all the operations of forming a needle rail type 60E1A6 with a shortened reforging length. As was expected, the highest forging force value, equaling about 2500 t, occurred in the last forging operation. The rail underwent significant cooling during the preceding forging operations and transport—on the corners of the forging, the temperature at the entrance into operation III was lower by over 300 °C than the initial temperature (1280 °C). It was the larger contact area during forming in operation III, the prolonged times of transport and the formed flash that caused the deformation force in the last operation to increase almost three times compared to operation II.

As we can notice, the maximal force needed to reforge this rail equals about 2400 t and is lower than the maximal pressure force of the press by about 600 t; therefore, we can assume that, in the case of technological trials under industrial conditions on the target hydraulic press with a pressure of 3000 t, there should be no problem with reforging and obtaining a proper forging. The obtained force values simultaneously confirm the results of physical modeling with the use of Pb and the recalculation of the forces from this experiment through the calculated scale factor C. At the same time, we should note that the simulation of forming a needle rail was based on the predetermined initial boundary conditions (e.g., temperature) and assumptions (e.g., friction coefficient = 0.15, determined as water with graphite in the Forge program database) that, in the case of a real experiment, can differ. However, an allowance of over 600 t, even in the case of increased friction forces, should be sufficient.

On this basis, it was stated that, based on the performed simulation tests confirmed with physical modeling and numerical simulations for the target process, we can make real metal tools.

**Figure 20:**
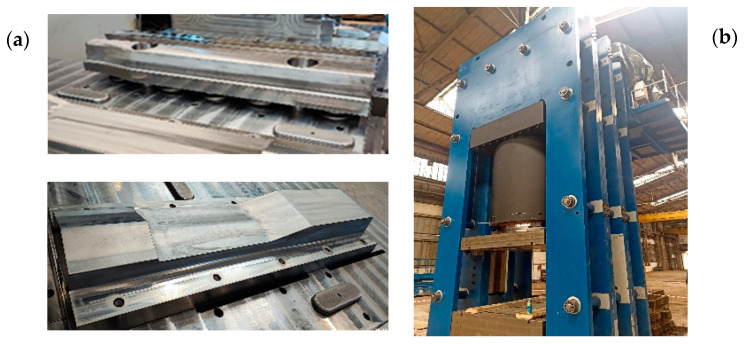
Photos of: (**a**) selected tools used to forge a 60E1A6 profile, (**b**) the hydraulic press of 5000 tons pressure.

We assumed that the tools, together with the remaining elements of forging instrumentation, would be made from tool steel for hot WLNV operations through milling and electrical machining. Currently, works are being performed connected with assembling the whole tool set and fixing the hydraulic press in the table (Figure 20).

## 4. Conclusions

This study presents the results of numerical and physical modeling that made it possible to build forging tools as well as develop guidelines for the created technology of hot precision forging needle rails used in railroad turnouts from profile 60E1A6 to 60E1. The first stage of investigations was the construction of a numerical FEM model as well as simulations of the process of forging a needle rail from lead in order to elaborate the preliminary geometry of the working impressions of the tools for physical modeling.

- The preliminary numerical simulation results for the process of forging from Pb referring to the force parameters demonstrate that verification of the numerical modeling would be conducted at 1:4 scale due to the obtained high forging force values (at 1:1 scale) as well as the availability of a semi-industrial hydraulic press with a nominal pressure of 200 t. The obtained results of numerical and physical modeling for a Pb rail are in good agreement, which is confirmed by the similar course of forging forces as well as their values in the case of the maximal forging force in numerical modeling (the determined error values equaled up to 8%);
- A similar comparison of the 3D scan images of the rail forged from lead to the CAD model obtained from FEM shows a good agreement, as the geometrical deviations are within the assumed tolerance of 0–0.15 mm. At the same time, we should emphasize that both in numerical modeling and physical modeling, certain simplifications were made, those being that the main assumed criteria were the mean deformation rate and temperature in the numerical modeling of the target process. Nevertheless, such an approach is justified in order to minimize the costs of the industrial experiment and with undertake attempts at developing the target technology under industrial conditions with the greatest probability of success;
- The final stage of research was the construction of a numerical model of the industrial forging process based on the physical modeling results, in particular re-scaling the geometry of the working impressions of the tools used to forge Pb. The obtained numerical simulation results for the target forging process, after recalculation of the forging forces from the physical modeling based on the similarity condition in the plastic scope and consideration of the 1:4 scale, demonstrate a good agreement in terms of forging force values;
- At present, works investigating mounting the tools in the holders on the press and selecting the optimal settings and technological parameters are being conducted, after which technological forging trials under industrial conditions will be realized. However, it is assumed that there might be a need for small process modifications and improvement of the technological parameters, e.g., adjusting the working temperature of the dies as well as the parameters and manner of lubrication, which of course is inevitable in the case of activating new forging processes;
- The results of the conducted research can serve in the future towards developing similar technologies for precision forging railroad turnout needle rails not only for the commonly used pearlitic steels, but also bainitic steels used for high-speed trains as well as other special applications.

## Data Availability

Data sharing not applicable.
